# Why Do Cancer Cells Become “Addicted” to Oncogenic Epidermal Growth Factor Receptor?

**DOI:** 10.1371/journal.pmed.0040321

**Published:** 2007-10-30

**Authors:** Ingo Mellinghoff

## Abstract

The author discusses three new studies in*PLoS Medicine* that shed light on the mechanisms involved in apoptosis triggered by EGFR kinase inhibitors.

## Targeting the Epidermal Growth Factor Receptor Kinase in Cancer

During the last five years, kinase inhibitors have emerged as a promising new class of cancer therapeutics [1]. These drugs target enzymes that are often ubiquitously expressed within the human body, control a wide range of cellular responses, and are tightly regulated under physiological conditions [2]. Cancer cells can escape these normal restraints on kinase activity through mutations in kinase-encoding genes or genes regulating their function [3]. Even though tumorigenesis is a multistep process requiring multiple genetic aberrations, cancer cells can become so dependent on, or “addicted” to, a deregulated signaling pathway that blocking this signal results in cell death [4]. Oncogene addiction was first documented in genetically engineered mouse models of cancer [5–7] and subsequently proven in the clinic by the dramatic response of BCR-ABL–positive leukemias to the small molecule ABL-kinase inhibitor imatinib [8,9].

Among kinase candidates to be targeted in epithelial cancers, the epidermal growth factor receptor (EGFR) was one of the first choices [10] based on the evidence in human tumor samples for oncogenic EGFR activation through *EGFR* gene amplification, gain-of-function deletions in the EGFR extracellular domain, and coexpression of EGFR and its ligands [11]. EGFR-targeted therapeutics have been explored in a large number of human malignancies and have shown clinical activity in subsets of patients with non-small cell lung cancer (NSCLC), glioblastoma, squamous cell carcinomas of the head and neck, colorectal carcinoma, and certain other malignancies [12]. The identification of *EGFR* kinase domain mutations in patients with NSCLC, and the association of these mutations with clinical responses to EGFR tyrosine kinase inhibitors (TKI), constituted a landmark discovery for our understanding of EGFR-mediated oncogenesis [13–15].

**Linked Research Articles**

This Perspective discusses the following new studies published in *PLoS Medicine:*

▪ Gong Y, Somwar R, Politi K, Balak M, Chmielecki J, et al. (2007) Induction of BIM is essential for apoptosis triggered by EGFR kinase inhibitors in mutant EGFR-dependent lung adenocarcinomas. PLoS Med 4(10): e294. doi:10.1371/journal.pmed.0040294
Using a panel of human drug-sensitive *EGFR* mutant lung cancer cells, William Pao and colleagues show that induction of BIM, a member of the BCL2 family, is essential for apoptosis triggered by EGFR kinase inhibitors.
▪ Costa DB, Halmos B, Kumar A, Schumer ST, Huberman MS, et al. (2007) BIM mediates EGFR tyrosine kinase inhibitor-induced apoptosis in lung cancers with oncogenic EGFR mutations. PLoS Med 4(10): e315. doi:10.1371/journal.pmed.0040315
Susumo Kobayashi and colleagues provide evidence that the polypeptide BIM is involved in tyrosine kinase inhibitor (TKI)-induced apoptosis in sensitive EGFR-mutant cells and suggest that induction of BIM may have a role in the treatment of TKI-resistant tumors.
▪ Cragg MS, Kuroda J, Puthalakath H, Huang DCS, Strasser A (2007) Gefitinib-induced killing of NSCLC cell lines expressing mutant *EGFR* requires BIM and can be enhanced by BH3 mimetics. PLoS Med 4(10): e316. doi:10.1371/journal.pmed.0040316
Andreas Strasser and colleagues demonstrate that activation of the proapoptotic BH3-only protein BIM is essential for tumor cell killing and that shutdown of the EGFR–MEK–ERK signaling cascade is critical for BIM activation.

Exactly how inhibition of EGFR signaling results in the often dramatic tumor responses of *EGFR*-mutant lung tumors has remained an enigma. Three highly complementary studies published in this issue of *PLoS Medicine* address this important question and identify the proapoptotic molecule BIM (BCL2-interacting mediator of cell death, also called BCL2-like 11) as critical mediator of EGFR TKI-induced cell death in EGFR-driven cancer [16–18].

## What the Three New Studies Show

To study the mechanisms of EGFR TKI-induced cell death, all three research teams took advantage of the large number of NSCLC cell lines that have been characterized in terms of their EGFR mutational status and cytotoxic response to the EGFR TKIs gefitinib and erlotinib: H3255, PC-9, and HCC827 cell lines showed the most dramatic apoptotic responses; H1975, A549, and H460 cells were resistant; and H1650 cells showed an intermediate response. Cell death in response to EGFR kinase inhibition featured cytochrome *c* release and activation of BAX and could be rescued by overexpression of BCL-x_L_, all consistent with activation of the mitochondrial “intrinsic” pathway of apoptosis.

Since activation of the intrinsic cell death pathway is governed by the balance between proapoptotic and antiapoptic BCL2 family members [19], the studies next looked for changes in the expression of BCL2 proteins that were most consistently correlated with the phenotype of EGFR TKI-induced apoptosis. Rapid dephosphorylation and increasing levels of the proapoptotic family member BIM, and in particular its splice variant BIM_EL_, was observed in all cell lines with a cytotoxic response. This correlation between BIM induction and EGFR TKI induced cell death was not limited to the in vitro environment as shown by Yixuang Gong and colleagues using two distinct transgenic mouse models of EGFR-driven lung cancer [16]. In contrast to the findings with BIM, changes in the expression of other “BH3-only proteins” (BAD, PUMA, and BMF), BAX family members (BAX and BAK), or antiapoptotic BCL2 family members (BCL2, BCL-x_L_, BCL-w, and MCL1) were not consistently associated with apoptosis.

Daniel Costa and colleagues further explored the relationship between BIM induction and EGFR TKI response in isogenic cell lines and showed that stable overexpression of a gefitinib-resistant *EGFR* allele (delE746-A750/T790M) in HCC827 cells markedly attenuated BIM induction and apoptosis in response to gefitinib [17]. BIM induction and apoptosis were restored in these cells when gefitinib was switched to the irreversible EGFR kinase inhibitor CL-387,785 which does inhibit the delE746-A750/T790M *EGFR* allele. Costa and colleagues also described a new *EGFR* mutation (L747S) in a tumor with acquired EGFR kinase inhibitor resistance and showed that IL3-independent Ba/F3 cells overexpressing this allele showed an intermediate degree of BIM1 induction and intermediate levels of apoptosis in response to gefitinib (i.e., relative to the hypersensitive L858R mutant and the resistant L858R/T790M mutant).

To prove the biological significance of BIM induction for EGFR TKI-induced cell death in EGFR-driven cells, all three groups demonstrated that RNA interference-mediated knockdown of *BIM* was sufficient to block or at least markedly attenuate the apoptotic response to EGFR kinase inhibition.

Using BH3 profiling [28] of mitochondria from EGFR mutant cells, Jing Deng et al. similarly identified BIM as critical effector of EGFR TKI induced cell death [29].

## Implications

Using RNAi technology and cells from gene-targeted mice, Junya Kuroda et al. recently demonstrated that BIM (and to lesser degree BAD) play a critical role in imatinib-induced apoptosis of BCR-ABL–positive leukemia cells [20]. The current studies demonstrate that activation of the intrinsic cell death pathway through induction of BIM is also required to elicit an apoptotic response to EGFR TKIs in NSCLC cells harboring mutant *EGFR*. While this observation remains to be formally proven in patients, it should be emphasized that the preclinical models used in these studies have a strong track record of predicting drug response and drug resistance in patients with NSCLC and *EGFR* mutations [13,21–24]. The current studies thus raise the question whether mutations in the intrinsic cell death pathway provide a mechanism of resistance to EGFR TKIs and perhaps other kinase inhibitors.

The level of BIM induction in *EGFR* mutant preclinical models appears to correlate with the extent of apoptosis and is influenced by drug concentration, *EGFR* genotype, and other unknown cellular factors. Since BIM functions by inhibiting BCL2 and its antiapoptotic homologs [25], it is perhaps not surprising that a BH3-mimetic compound (ABT-737), which binds to BCL2, BCL-x_L_, and BCL-w, can synergize with low-dose EGFR kinase inhibitor therapy to induce apoptosis in EGFR-driven cells as shown by the Gong and Cragg groups [16,18]. This latter finding suggests that combining EGFR TKIs with a BH3-mimetic peptide might provide additional benefit over EGFR TKI monotherapy in tumors with questionable drug penetration or certain *EGFR* genotypes.

**Glossary:**

**BCR-ABL:** Fusion of the BCR (breakpoint cluster region) gene on Chromosome 22 to the ABL (Ableson leukemia virus) gene on Chromosome 9 is found in 95% of patients with chronic myeloid leukemia. The resultant fusion protein BCR-ABL is a constitutively active cytoplasmic tyrosine kinase and the molecular target of the small molecule kinase inhibitor imatinib (Gleevec).

**The BCL2 protein network:** The family of BCL2 (B cell lymphoma 2)-related proteins in mammals includes at least 20 members that compete with each other to promote or block cell death in response to various forms of stress. BCL2-related proteins are usually grouped into three distinct categories: the antiapoptotic proteins (BCL2, BCL-x_L_, MCL1, BCL-w, and A1/Bfl-1), the proapoptotic proteins (BAX and BAK), and the “BH3-only proteins” (BIM, BAD, PUMA, NOXA, BIK, and BID), which share a single motif (a BCL2-homology 3 [BH3] domain) with other BCL2 proteins. BH3-only proteins promote cell death by inhibiting antiapoptotic BCL2 family members, which themselves restrain BAX and BAK activity. BAX and BAK activation result in permeabilization of the mitochondrial membrane, cytochrome *c* release, and the activation of caspases, which execute the cell-suicide program. The *BIM* mRNA undergoes alternative splicing to give rise to the short, long, and extra-long protein variants (BIM_S_, BIM_L_, and BIM_EL_).

**Ba/F3 cells:** Murine Ba/F3 pro-B lymphocytes provide a unique model system with which to examine kinase inhibitor sensitivity because stable expression of oncogenic kinases can relieve these cells from their intrinsic dependence on interleukin 3 (IL3) for survival. The Ba/F3 cells do not express endogenous *EGFR* and thus provide an opportunity to compare ectopically expressed *EGFR* mutants within the same cellular background.

The Gong and Cragg groups show that inhibitors of the EGFR effector kinase MEK1/2 phenocopy the effects of EGFR TKIs on BIM, but fail to trigger apoptosis [16,18]. These data suggest that BIM-independent EGFR signals are required to cause cell death in *EGFR*-mutant NSCLC cells. Additional studies are needed to identify these signals and determine how specific oncoproteins modulate BIM and other BCL2 family members at the transcriptional and post-translational level [26,27]. By identifying critical effectors of kinase inhibitor–induced cell death, the recent studies have brought us one big step closer to unraveling the mysteries of oncogene addiction.

## Abbreviations

BIM: BCL2-interacting mediator of cell death
EGF: epidermal growth factor
EGFR: epidermal growth factor receptor
NSCLC: non-small cell lung cancer
TKI: tyrosine kinase inhibitor

## Note Added in Proof

References 28 and 29 are cited out of order because they were added when the article was in proof.
